# Compound DNA vaccine encoding SAG1/ SAG3 with A_2_/B subunit of cholera toxin as a genetic adjuvant protects BALB/c mice against *Toxoplasma gondii*

**DOI:** 10.1186/1756-3305-6-63

**Published:** 2013-03-13

**Authors:** Hua Cong, Min Zhang, Qing Xin, Zhiyu Wang, Ying Li, Qunli Zhao, Huaiyu Zhou, Shenyi He

**Affiliations:** 1 Department of human parasitology, Shandong University School of Medicine, No44 wenhuaxi Road, Jinan, Shandong, 250012, P. R. China; 2 School hospital of Shandong University, Jinan, Shandong, 250012, P. R. China; 3 School of Public Health, Shandong University, No44 wenhuaxi Road, Jinan, Shandong, 250012, P. R. China

**Keywords:** Toxoplasma gondii, Surface antigen, SAG1, SAG3, CTXA_2_/B, DNA vaccination

## Abstract

**Background:**

Intracellular parasites, such as *T*. *gondii*, present a plurality of antigens because of the complexity of its life cycle. Compound DNA vaccines bring a new approach and hope for the treatment of toxoplasmosis. In this study, a DNA vaccine encoding two major surface antigens SAG1, SAG3 from *T. gondii*, with A_2_/B subunit of cholera toxin as a genetic adjuvant was constructed.

**Methods:**

BALB/c mice were immunized intramuscularly with PBS, pcDNA3.1, pSAG1, pSAG1/SAG3 and pSAG1/SAG3-CTXA_2_/B three times separately. Immunized mice were tested for IgG antibody and IFN-γ and IL-4 production by ELISA. The proliferation of T cells was measured by DNA synthesis assay and the lymphocyte subsets of spleen cells by flow cytometry. All the immunized mice were challenged with 10^3^ highly virulent RH tachyzoites of *Toxoplasma gondii* intraperitoneally and the survival times were recorded.

**Results:**

An enhanced production of IgG antibodies, antigen-specific lymphocyte proliferation and IFN-γ production from splenic cells were induced in mice immunized with pSAG1/SAG3 compared to mice immunized with pSAG1 (P<0.05). Introduction of CTXA_2_/B further enhanced the Th1 cell-mediated immunity with higher levels of IFN-γ, lymphocyte proliferation activity and percentage of CD8^+^ T-cells. When challenged with lethal doses of *T. gondii* (1×10^3^), all control mice (PBS and empty plasmid group) died within 6 days. Mice immunized with pSAG1 died within 8 days. While 20% and 40% survival rate were achieved from mice immunized with pSAG1/SAG3 and pSAG1/SAG3-CTXA_2_/B.

**Conclusions:**

This study indicates the compound DNA vaccine encoding *T. gondii* antigens SAG1, SAG3 with CTXA_2_/B gene was a promising DNA vaccine candidate against toxoplasmosis, which could effectively enhance the humoral and cellular immune response and prolong survival time in vaccinated mice.

## Background

*Toxoplasma gondii* is a single-cell obligate intracellular protozoan, which is widely prevalent all over the world. Prevalence of *T. gondii* infection increased by 7% during the past ten years in China [1]. This parasite is of major medical importance, being a cause of congenital disease and abortion [2]. In immunocompromised patients, such as those with cancer or AIDS, the disease can be fatal [3,4]. Development of an effective vaccine is an attractive way to prevent this disease.

In recent years, *T. gondii* vaccines have made great progress from the earlier mutant strains to the latest DNA vaccine [5-9]. Especially compound polyvalent DNA vaccines bring about a new approach and hope for *T. gondii*. Because complex intracellular parasites, such as *T. gondii*, present a plurality of antigens and as the antigen presentation capability varies widely among different individuals, immunization with a vaccine that includes a broad array of antigens is likely to be more efficacious than a single antigen.

The surface of the tachyzoite is the main target of the host immune response. The tachyzoite surface is dominated by SAG1, SAG2A, SAG3, SRS1, SRS2, and SRS3 [10]. Examples of immunization experiments with *T. gondii* DNA encoding SAG antigens, alone or in combination with other antigens have already been reported [11-13]. SAG1 and SAG3 share an overall similar folding, which was shown to participate in the cellular invasion by the parasite [14,15]. The SAG1 gene, encoding *T. gondii* P30 protein and accounting for 5% of all proteins in the tachyzoite, is the first tachyzoite antigen to be cloned and sequenced, which enables invasion of host cells by binding to cellular receptors [16]. This protein links the parasite and host cell receptor, which favours parasite invasion of host cells [17]. SAG3 is the first glycoaminoglycan-binding protein associated with *Toxoplasma*, and SAG3–heparan sulfate proteoglycans (HSPGs) interactions are involved in the parasite attachment to target cells [18]. A previous study clearly demonstrated that SAG3 is important for the parasite adhesion to host cells [19], which was considered as one member of the receptors system of *T. gondii* that act as ligands mediating host cell recognition and attachment. Although SAG3 is very similar to SAG1 in structure and function, few studies have been performed with SAG3.

In this study, we constructed a DNA vaccine expressing two major surface antigens SAG1, SAG3 from *T. gondii*, with the A_2_/B subunit of cholera toxin as a genetic adjuvant. The immunity induced by this DNA vaccine in BALB/c mice and the protection afforded against challenge with the highly virulent RH strain of *T. gondii* is evaluated.

## Methods

### Parasites and soluble tachyzoite antigens

The tachyzoites of the highly virulent RH strain of *T. gondii* were stored in liquid nitrogen in our laboratory. The parasites were maintained by serial intraperitoneal passage in BALB/c mice. The tachyzoites were harvested from the peritoneal fluid of mice after 72 h, and used for genomic DNA extraction, the vaccine challenge infection study and soluble tachyzoites antigens extraction.

The peritoneal fluid was washed by 0.01M phosphate buffered saline (PBS) three times in a low speed centrifugation and disrupted using an ultrasonic disintegrator, followed by freezing and thawing (six cycles), and then centrifuged at 1500×g for 15 min. The supernatant containing soluble tachyzoites antigens (STAg) was kept at −20°C until further use.

### Plasmids construction

Three pairs of primers were designed and synthesized according to the published gene sequence of *T. gondii* (RH strain) and the A_2_/B subunit of cholera toxin. Restriction endonuclease sites were added at the 5^′^ ends of sense and antisense strands of the primers, respectively, to allow SAG1 gene, SAG3 gene and CTXA_2_/B gene orientation and to ensure the precision of the opening reading frame.

SAG1 primer: forward 5^′^-CGGAATTCATGACGGAGAACCACTTCACTC-3^′^, reverse 5^′^-ATGGATCCCGCGACACAAGCTGCGATAG-3^′^; SAG3 primers: forward ’-GGATC GGA TCC ATGCAGCTGTGGCGGCGCAGAGC-3^′^, reverse 5^′^-TGATCGGTACCTTTCTGTTCCAGCTTGACTTTCC- 3^′^; CTXA_2_/B primers: forward 5^′^- CG GGT ACC AGT AAT ACT TGC GA- 3^′^, reverse 5^′^- AC AAGCTTTTA ATT TGC CAT AC-3^′^ .

**Figure 1:**
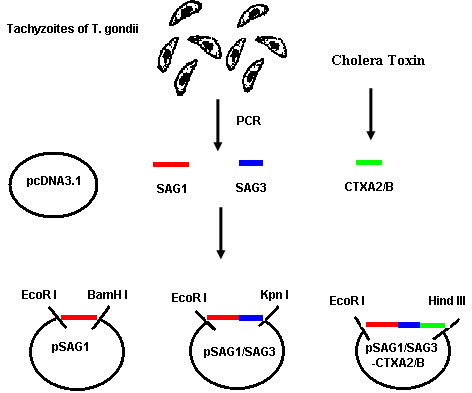
**The schematic diagram of the construction of DNA vaccines.** SAG1 gene, SAG3 gene of *T. gondii* and CTXA_2_/B gene of cholera toxin were introduced into the eukaryotic expression plasmid pcDNA3.1 (−) vector by EcoR I / BamH I, EcoR I / Kpn I or EcoR I / Hind III cloning sites.

The compound gene was obtained by T-A cloning (TaKaRa, Dalian) and introduced into the eukaryotic expression plasmid pcDNA3.1 (−) vector by EcoR I/ BamH I, EcoR I/Kpn I or EcoR I/Hind III cloning sites separately. The construction of DNA vaccines was shown in Figure 1. *Escherichia coli* DH5α cells were transformed with the ligation mixture by calcium chloride. The recombinant plasmids pSAG1, pSAG1/SAG3 and pSAG1/SAG3-CTXA_2_/B with the correct insert orientation was detected by restriction enzymes analysis, PCR and then purified by a column chromatography kit (Omega, USA) and sequenced (Bioasia, Shanghai).

### Expression of compound gene *in vitro*

The recombinant eukaryotic expression plasmids pSAG1, pSAG1/SAG3 and pSAG1/SAG3-CTXA_2_/B were transfected into HeLa cells by liposomes (LipofectAMINE™ 2000, Invitrogen) and stable strains of transfectants were obtained after being screened by G418 (Gibco, BRL). The genes that input in the plasmid pcDNA3.1 were verified to have the capability to transcript *in vitro* by RT-PCR.

### Immunization of BALB/c mice

SPF BALB/c female mice (6–8 weeks old) were used in all the immunization and parasite challenge experiments. They were purchased from Shandong University Laboratory Animal Center and maintained under standard conventional conditions. All studies were conducted with approval from the Institutional Animal Care and Use Committee at the University of Shandong.

Large scale recombinant plasmid DNA was prepared by the alkaline lysis method. Plasmids were diluted and suspended in sterile phosphate buffered saline (PBS) to a final concentration of 1 μg/μl. BALB/c mice were randomly divided into five groups (20 mice/each group). Three experimental groups of mice were injected with 100 μl of 1 μg/μl plasmid pSAG1, pSAG1/SAG3 and pSAG1/SAG3-CTXA_2_/B separately in the quadriceps muscle. Two control groups received 100 μg of empty vector pcDNA3.1 or 100 μl PBS (Phosphate buffered saline). Booster immunizations were administered twice at 2 week intervals. Mice were immunized on days 0, 14 and 28 and the tail vein serum samples were collected on the day before immunization and 2, 4, 6 weeks after immunization.

### Evaluation of humoral responses

To measure *T. gondii*-specific IgG, plates (Dursley, UK) were coated overnight with 10 μg/ml soluble tachyzoite antigen in 0.1 M carbonate buffer pH 9.6 (50 μl per well). After two hours’ blocking, sera diluted 1:200 in 1% BSA–PBST20 (50 μl per well) were added and incubated for 1 h at RT. After washing, bound antibodies were detected by incubation at RT for 1 h with horseradish peroxidase (HRP)-conjugated goat anti-mouse immunoglobulins IgG, (Pharmingen, USA) at 1:1000 dilution in 1% BSA–PBST20 (50 μl per well). Peroxidase activity was revealed by adding 50 μl per well of a solution containing 12.5% H_2_O_2_, 0.1 M citrate-phosphate (pH 4) and 10 mg/ml of 3, 3^′^, 5, 5^′^- tetramethylbenzidine (TMB). The reaction was stopped by adding 50 μl of 2 M H_2_SO_4_ and the optical density (OD) was read out at 492 nm in an ELISA microplate reader (Bio-TEK, USA).

### Lymphocyte proliferation assays

Three mice from each group were euthanized two weeks after the last immunization. Their spleens were removed under sterile conditions. Single-cell suspensions were obtained by filtration through nylon mesh. After removal of the erythrocytes, the remaining spleen cell suspensions were adjusted to a final concentration of 5×10^6^ cells/ml in complete RPMI 1640 (Gibco-BRL) tissue culture medium. Splenocyte suspensions (100 μL per well) were plated into 96-well U-bottomed tissue culture plates along with 100 μl of stimulant diluted to appropriate concentrations in complete RPMI 1640. The stimulant used was *T. gondii* tachyzoite antigen at 50 μg/ml. Concanavalin A (10 μg/ml, Sigma) was used as a positive control, and cells cultured with media alone were used as negative controls. The plates were incubated for three days in 5% CO_2_ at 37°C and pulsed with 1 μ Ci [^3^H] thymidine per well for the final 18 h. The cells were then harvested onto glass fiber filters using a cell harvester (Skatron Instruments, Norway). The radioactivity incorporated into the DNA was determined by liquid scintillation.

### Flow cytometry (FCM) of T lymphocyte subsets

Two weeks after immunization, the spleen cells were isolated from immunized mice with RMPI 1640 medium without serum. Cells adjusted to 1 × 10^6^ with PBS were stained with FITC-conjugated anti-mouse CD8^+^ monoclonal antibody (eBioscience, USA) and PE- conjugated anti-mouse CD4^+^ monoclonal antibody (eBioscience, USA), T lymphocyte subsets were measured using flow cytometry (Beckman Coulter, USA).

### Cytokine assays

Splenocytes from immunized mice were cultured with STAg as described for the lymphocyte proliferation assay. At the end of the incubation period, the culture supernatants were harvested and centrifuged for 5 min at 110×*g*. Commercial ELISA kits (mouse IFN-γ OptEIA, IL-4 OptEIA, Endogen, USA) were used according to the manufacturer’s instructions to assay cytokine levels in culture supernatants obtained at 24 h for IL-4 and 96 h for IFN-γ.

### Challenge infection

Ten mice in each group were challenged intraperitoneally with 10^3^ tachyzoite forms of *T. gondii* RH strain 4 weeks after the last immunization. The mice were observed for 30 days and the time to death was recorded where appropriate.

### Statistical analysis

The statistical significance between different groups was calculated with one-factor analysis of variance (ANOVA). Differences were considered to be significant with P < 0.05.

## Results

### Plasmid construction and *in vitro* expression of compound genes

**Figure 2:**
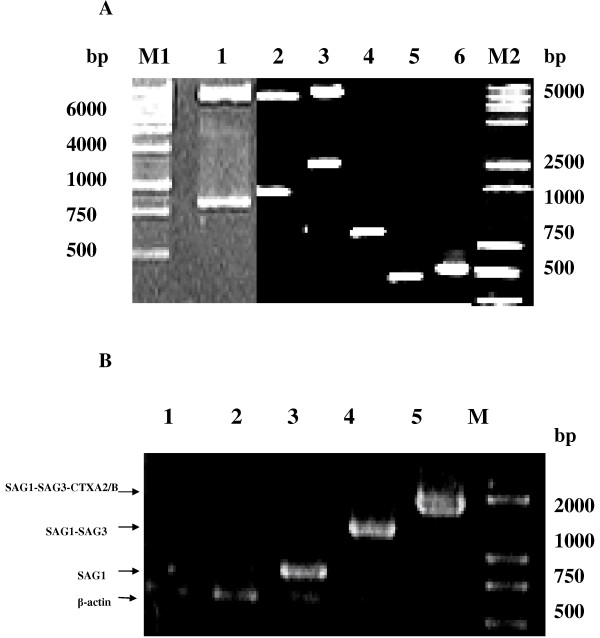
**The identification of recombinant plasmids and *in vitro *expression of compound genes.A**: Identification of recombinant plasmids pSAG1; pSAG1/SAG3; pSAG1/SAG3-CTXA_2_/B. Lane M1: DNA Marker 6000 Lane1. pSAG1 digested by EcoR I and BamH I; Lane2. pSAG1/SAG3 digested by EcoR I and Kpn I; Lane3. pSAG1/SAG3-CTXA2/B digested by EcoR I and Hind III; Lane 4. PCR product of SAG1; Lane 5. PCR product of SAG3; Lane 6. PCR product of CTXA2/B; Lane M2: DNA Marker 5000. **B**: RT-PCR results of Hela cell transfected by recombinant plasmid. Lane 1, 2, 3, 4, 5: Hela cells transfected by Liposome, pcDNA3.1(−), pSAG1, pSAG1/SAG3, pSAG1/SAG3-CTXA2/B respectively; Lane M DNA Marker 2000.

The recombinant plasmids pSAG1, pSAG1/SAG3 and pSAG1/SAG3-CTXA_2_/B with the correct insert orientation were detected by restriction enzyme analysis and PCR. The sizes of SAG1, SAG3, CTXA_2_/B were 786bp, 492bp, 512bp respectively (Figure 2A).

pSAG1, pSAG1/SAG3 and pSAG1/SAG3-CTXA_2_/B plasmids transfection bands were at 786bp, 1278bp, 1790bp respectively, which proved all the genes could be expressed in HeLa cells. While there are only β-actin gene bands in HeLa cells transfected by Liposome and pcDNA3.1 (−) (Figure 2B).

### Evaluation of humoral responses

**Figure 3:**
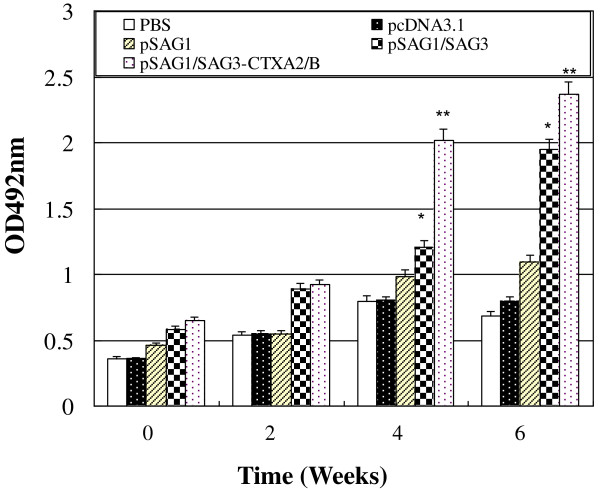
**Kinetics and strength of humoral response in BALB/c mice immunized with PBS, pcDNA3.1, pSAG1, pSAG1/SAG3, pSAG1/SAG3-CTXA**_**2**_**/B.** Mice were immunized on days 0, 14 and 28 and the tail vein serum samples were collected on the day before immunization and 2, 4, 6 weeks after immunization.

BALB/c mice intramuscularly immunized with PBS, pcDNA3.1 or pSAG1 resulted in none or only low anti-*T. gondii* IgG titres in the serum of mice. In contrast, much higher levels of anti-*T. gondii* IgG antibodies were detected when mice were immunized with pSAG1/SAG3 compared to pSAG1 (P<0.05). When CTXA_2_/B genetic adjuvant was included, anti- *T. gondii* IgG values increased markedly in the pSAG1/SAG3-CTXA2/B immunized group, which were significantly higher than those of negative controls (P<0.01). There was a significant difference in anti-*T. gondii* IgG antibodies between mice immunized with or without CTXA_2_/B as a genetic adjuvant (P<0.05) (Figure 3).

### Cellular immune response analysis

Culture supernatants from antigen-stimulated splenocytes were quantified with sandwich ELISA for Th1- and Th2-type cytokines. Table 1 shows pSAG1, pSAG1/SAG3 and pSAG1/SAG3-CTXA_2_/B immunization groups could induce splenic T cells to secret high levels of IFN-γ compared to control groups immunized with PBS and pcDNA3.1 (P<0.05). Double genes could induce splenic cell to secret significant higher level IFN-γ production in immunized mice than a single gene (P<0.05). Cultured splenocytes from pSAG1/SAG3 and pSAG1/SAG3-CTXA_2_/B vaccinated mice demonstrated a preferential production of IFN-γ on stimulation with tachyzoite antigens, with little production of IL-4, suggesting that the response was oriented to a Th1 type. Similarly, antigen-specific lymphocyte proliferation in pSAG1/SAG3 immunization mice were higher than pSAG1 immunized mice (P<0.05). CTXA_2_/B as a genetic adjuvant could effectively enhance the lymphocyte proliferation activity in cultured splenocytes from pSAG1/SAG3-CTXA_2_/B vaccinated mice (P<0.01).

**Table 1:** Measurement of the cytokines in the immunized mice by sandwich ELISA

**Immunization regimen**^**a**^	**Cytokine production (pg/ml)**^**b**^		**SI**^**c**^
	**IFN-γ**	**IL-4**	
PBS	11±5	N.D	0.17
pcDNA3.1	13±4	N.D	0.32
pSAG1	24±6	N.D	1.38
pSAG1/SAG3	47±7	N.D	2.47
pSAG1/SAG3-CTXA_2_/B	89±10	N.D	4.28

^a^ Mice were immunized by i.m. route on day 0 and day 14 and day 28 with PBS, pcDNA3.1, pSAG1, pSAG1/SAG3, pSAG1/SAG3-CTXA_2_/B.

^b^ The splenocytes taken from mice (n = 3, each group) 2 weeks after the last immunization were examined for cytokine production by sandwich ELISA. Values for IFN-γ from 96 h, values for IL-4 from 24 h. N.D means not detectable.

^c^ The results of proliferation assays are expressed as the stimulation index (SI), calculated as the ratio between the mean counts per minute (cpm) for triplicate stimulated cultures and the mean counts per minute for triplicate unstimulated cultures. SI values 2.5-fold greater than the SI of the control groups were considered as significant.

To evaluate the change in T cell subsets after immunization, we performed a phenotype analysis of murine splenocytes. CD4^+^ and CD8^+^ T lymphocyte subsets from the immunized mice were assayed by flow cytometry. Table 2 shows the percentage of CD4^+^ and CD8^+^, and the ratios of CD4^+^ and CD8^+^ T-cells. The percentage of CD4^+^ T-cells in immunization group did not change significantly compared to the control group. However, the percentage of CD8^+^ T cells in immunization group changed significantly. In mice immunized intramuscularly with pSAG1, the percentage of CD8^+^ T cells is 26.37 ± 0.98, which is higher than the CD8^+^ T cells percentage in PBS group (18.67 ± 0.92), but not significant different compared to empty plasmid pcDNA3.1 group. In mice group immunized with pSAG1/SAG3 or pSAG1/SAG3- CTXA_2_/B plasmid, the percentage of CD8^+^ T cells was 35.01 ± 0.88, 38.55 ± 0.94 and greatly increased compared to other groups. Conversely, the ratios of CD4^+^ and CD8^+^ T-cells decreased.

**Table 2:** **CD4**^**+**^**CD8**^**+**^**subtype of T cells from immunization mice were measured using flow cytometry**

**Immunization regimen**^**a**^	**CD4**^**+**^**(%)**^**b**^	**CD8**^**+**^**(%)**^**b**^	**CD4**^**+**^**/CD8**^**+**^
PBS	26.71±0.22	18.67±0.92	1.43±0.23
pcDNA3.1	27.62±0.25	24.71±0.85	1.12±0.29
pSAG1	26.45±0.34	26.37±0.98	1.00±0.35
pSAG1/SAG3	24.68±0.14	35.01±0.88	0.70±0.16
pSAG1/SAG3-CTXA2/B	25.15±0.23	38.55±0.94	0.65±0.24

^a^ Mice were immunized by i.m. route on day 0 and day 14 and day 28 with PBS, pcDNA3.1, pSAG1, pSAG1/SAG3, pSAG1/SAG3-CTXA_2_/B.

^b^ The splenocytes culture supernatants taken from mice (n = 3, each group) 2 weeks after the last immunization were stained with FITC-labeled anti-mouse CD8^+^ monoclonal antibody and PE-labeled anti-mouse CD4^+^ monoclonal antibody, T lymphocyte subsets were measured using flow cytometry.

### Challenge study

**Figure 4:**
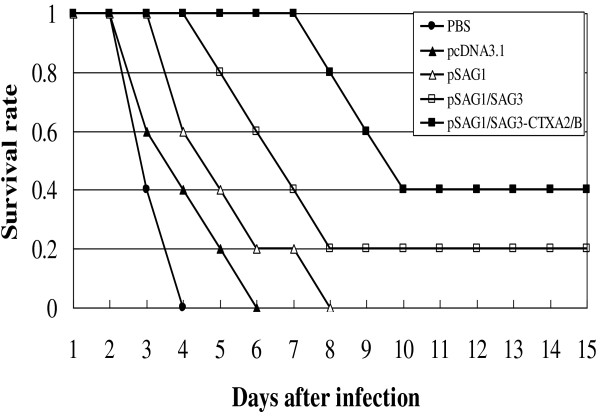
**Survival rates of immunized mice after challenging with RH tachyzoites of *T. gondii*.** BALB/c mice were immunized with PBS(● ), pcDNA3.1 (▲), pSAG1(Δ), pSAG1/SAG3(□), pSAG1/SAG3-CTXA_2_/B (■) three times with two weeks interval. Four weeks after final inoculation, mice (10 per group) were administered by challenging intraperitoneally with 1 x 10^3^ tachyzoites of *T. gondii* RH strain.

Four weeks after immunization, mice were challenged with 100 μl 1 × 10^3^ RH strain tachyzoites of *T. gondii* intraperitoneally. Mice which were treated with PBS died within 4 days. Mice immunized with empty plasmid intramuscularly died within 6 days. Mice immunized with pSAG1 died within 8 days. While there was a 20% survival rate for the mice immunized with pSAG1/SAG3 plasmid intramuscularly. Furthermore, pSAG1/SAG3-CTXA_2_/B vaccinated mice not only survived longer but 40% of the mice survived (Figure 4).

## Discussion

In this study, a DNA vaccine expressing two major surface membrane antigens SAG1, SAG3 from *T. gondii*, with or without the A2/B subunit of cholera toxin as a genetic adjuvant was constructed. The immunity induced by this vaccine in BALB/c mice and the protection afforded against challenge with the highly virulent RH strain of *T. gondii* was evaluated.

Our results demonstrate that *T. gondii* surface membrane antigens SAG1 and SAG3 genes, when used as a multivalent DNA vaccine are capable of inducing a stronger immune response than SAG1 single gene DNA vaccine. Numerous studies have supported the role of SAG1 in protection against *T. gondii* infection [20-22]. These studies show vaccination with SAG1 DNA could induce specific humoral and cellular immune responses, survival prolongation and brain cyst reduction. In another study, a Glutathione-s-transferase (GST)-fused SAG3 of *T. gondii* (rSAG3) was used to immunize BALB/c mice alone or in combination with Quil A (rSAG3/Quil A), which resulted in partial protective immunity against *T. gondii* infection through induction of a Th1-type immune response [23].

In this study, a significant production of IgG antibodies that recognize total *T. gondii* antigen was induced in mice immunized with pSAG1/SAG3 compared with mice immunized with pSAG1 (P<0.01). Similarly, antigen-specific lymphocyte proliferation of splenocytes from mice in the pSAG1/SAG3 immunization group were higher than pSAG1 immunization group (P<0.05). Also the double gene group could induce T cells to secret high levels of IFN-γ production compared to the single gene group. Furthermore, a high level of protection was achieved in the double gene group. The mice immunized with pSAG1/SAG3 not only showed extended survive times but maintained a 20% survival rate after challenge with 10^3^ RH tachyzoites, while all the pSAG1 immunization group died within eight days after challenge. Thus indicates that multi-antigenic DNA vaccines provide better protection against toxoplasmosis and are superior to a single-antigen DNA vaccine.

Further, in order to enhance the immunogenicity of the vaccine, the multivalent antigen gene was connected to the A_2_/B subunit of cholera toxin. CTXA_2_/B, A_2_/B subunit of a cholera toxin, has been demonstrated to be an effective adjuvant in our previous study [24,25]. In this study, CTXA_2_/B was linked to the double gene vaccine construct as a genetic adjuvant. As expected, stronger immune responses were induced by this vaccine in BALB/c mice and even high levels of protection were afforded against challenge with the highly virulent RH strain. Enhanced IgG antibodies were observed in pSAG1/SAG3-CTXA_2_/B immunization group in week 4 and week 6. For the cellular immune response the difference was significant (P<0.05) between groups with and without CTXA_2_/B gene. CTXA_2_/B gene adjuvant greatly improves the secretion of IFN-γ and lymphocyte proliferation activity, especially in the pSAG1/SAG3-CTXA_2_/B immunization group to maintain a 40% survival rate after challenge with 10^3^ RH tachyzoites.

CD4^+^ and CD8^+^ T-cell subsets play a central role in the establishment of protective immunity in the host and are largely involved in the protection provided by vaccines [26,27]. CD8^+^ T-cell is a major cellular T cell subset involved in acquired immune protection against *T. gondii*. There was a great increase in the CD8^+^ T lymphocytes subsets percentage in the pSAG1/SAG3 and pSAG1/SAG3-CTXA_2_/B immunization mice group, with the ratio of CD4^+^ T-cells and CD8^+^ T-cell in mononuclear cells showing significant differences compared to the control and pSAG1 immunization mice. Both T-cell subsets are important sources of IFN-γ, more optimum protective CD8^+^ T cell responses depend on the ability of CD4^+^ T-cells to provide the growth factor IL-2 [28,29]. Herein, compared with multivalent DNA vaccine immunization, introduction of CTXA_2_/B further enhanced the Th1 cell-mediated immunity with higher levels of IFN-γ but low levels of IL-4. These results clearly demonstrate that CTXA_2_/B can significantly augment Th1-type cellular immune responses in BALB/c mice.

In order to evaluate of protection potency, highly virulent RH strain of *T. gondii* was used for the challenge study. No effective vaccine has been shown to completely protect against intraperitoneal challenge with the RH strain of *T. gondii*[30,31]. In our study, when challenged with lethal doses of *T. gondii* (1×10^3^), all control mice (PBS and empty plasmid group) died within 6 days. Low protection against RH strain challenge was observed in pSAG1 immunization group. While there was a 20% survival rate for the mice immunized with pSAG1/SAG3 plasmid intramuscularly. Furthermore, pSAG1/SAG3-CTXA_2_/B vaccinated mice not only survived longer but showed a 40% survival rate. For the evidence for the differential expression of miRNAs in the two genetically distinct strains, RH and ME49, of *T. gondii* has been identified and defined [32]. The efficacy of this compound DNA vaccine evaluated by comparison of the brain tissue cyst burden in vaccinated and control groups, using a moderately virulent *T. gondii* strain, ME49, might be performed in the future [33].

## Conclusions

Multi-component DNA vaccine encoding *T. gondii* antigens SAG1, SAG3 with the adjuvant CTXA_2_/B gene could enhance the humoral and cellular immune response accompanied by a significant increase in survival rates in vaccinated mice. CTXA_2_/B as a genetic adjuvant could enhance humoral and cellular immunity of DNA vaccines. This compound DNA vaccine is a promising candidate to protect animals and humans against *Toxoplasma gondii*.

## Competing interests

The authors declare that they have no competing interests with this publication.

## Authors’ contributions

HC carried out the vaccine construction and drafted the manuscript. MZ carried out the plasmid construction, cultivated parasites and drafted the figures in the manuscript. QX and ZW performed the statistical analysis. YL and QZ performed the animal experiments. SH and HZ participated in the design of the study and manuscript revision. All authors have read and approved the final manuscript.
